# Dickkopf-related protein 3 alters aerobic glycolysis in pancreatic cancer BxPC-3 cells, promoting CD4^+^ T-cell activation and function

**DOI:** 10.1186/s40001-021-00567-x

**Published:** 2021-08-14

**Authors:** Qingqu Guo, Yiming Chu, Hongbo Li, Dike Shi, Lele Lin, Weifeng Lan, Dan Wu

**Affiliations:** 1 Department of Gastrointestinal Surgery, The Second Affiliated Hospital, College of Medicine, Zhejiang University, No. 88 Jiefang Road, Hangzhou, 310009 Zhejiang China; 2 Department of Gastrointestinal Surgery, The First Affiliated Hospital of Zhejiang Chinese Medicine University, Hangzhou, Zhejiang China; 3 Department of Surgery, Suichang County Hospital, No. 143 North Street, Suichang County, Lishui City, 323300 Zhejiang China

**Keywords:** Dickkopf-related protein 3, Pancreatic cancer, β-catenin, CD4^+^ T cells, Aerobic glycolysis

## Abstract

**Background:**

To investigate the value of Dickkopf-related protein 3 (DKK3) on aerobic glycolysis in pancreatic cancer cells, where DKK3-overexpression is used to determine its effects on CD4^+^ T cells.

**Methods:**

The BxPC-3-DKK3 cell line was constructed, and peripheral blood mononuclear cell (PBMC) was prepared. After isolated the CD4^+^ T cells, the lactic acid, glucose uptake ability, cellular viability, proliferation, apoptosis, and markers were detected by PCR and western blot, and the concentrations of multiple cytokines were determined using the ELISA method.

**Results:**

After co-culture with pancreatic cancer cells overexpressing DKK3, the glucose uptake markedly, proliferation enhanced and apoptosis inhibited in CD4^+^ T cells. The co-culture model also revealed that DKK3-overexpression promotes the activation and regulates the metabolism and function of CD4^+^ T cells.

**Conclusions:**

DKK3 alters the metabolic microenvironment of pancreatic cancer cells and further facilitates the function of CD4^**+**^

T cells which suggesting that DKK3 may have a therapeutic potential in pancreatic cancer.

## Background

As one of the aggressive malignancies, pancreatic cancer had poor prognosis and only 10–15% of patients were suitable for surgery. Although systemic chemotherapies, such as gemcitabine, have contributed to disease control and improved the clinical outcome, significant progress in other therapeutic areas has been limited [1]. As cancer cells can disengage the immune response via metabolic regulation, successful immunotherapy for pancreatic cancer remains elusive.

Therefore, discovering novel methods for improving T-cell function is of critical importance. As the primary nutrient source for adenosine triphosphate (ATP), glucose is necessary for T-cell activation, proliferation, and cytokine production [2]. T-cell activation is thought to drive the initial increase in glucose uptake, which is accompanied by an upregulation in aerobic glycolysis [3]. However, when glucose and glycolysis rates are low, prolonged periods with inadequate nutrients may result in T-cell energy [4].

Hypoxia is a risk factor for pancreatic cancer, because pancreatic cancer cells must adapt to a metabolically challenging environment during hypoxia [5]. Therefore, pancreatic cancer cells with increased glucose consumption (as a result of aerobic glycolysis) may promote glucose deprivation and subsequent acquisition of an anergic phenotype [3].

As a member of the human dickkopf family, DKK3 exhibits divergent characteristics in malignant tumors and is also an important regulator of Wnt signaling in tumors [6, 7]. However, the value of DKK3 in pancreatic cancer cells remains unknown.

In this study, we hypothesized that DKK3 may affect the proliferation and function of CD4^**+**^ T cells by regulating glucose metabolism in pancreatic cancer cells to investigate the value of DKK3 on aerobic glycolysis in pancreatic cancer cells.

## Methods

### Cell culture and hypoxia

In this study, we used human pancreatic adenocarcinoma cell line BxPC-3 which came from the American Type Culture Collection. The BxPC-3-DKK3 cell line was constructed according to our previous research [8]. RPMI 1640 medium supplemented with 10% fetal bovine serum were used for cells culture (37 °C, 5% CO_2_). Besides, we used 10 μg/ml mitomycin (MilliporeSigma) pretrested the BxPC-3 cells for 2 h to inhibit expansion and ensure that the appropriate concentration of tumor-to-T cells was maintained. For co-cultivation, CD4^+^ T cells were first seeded into the lower chamber of a co-cultivation system for adherence, while BxPC-3 or BxPC-3-DKK3 cells were seeded into the upper chamber. After stimulated with 1 μg/ml anti-CD3 antibody (Proteintech, 17,617-1-AP) and 0.5 μg/ml anti-CD28 antibody (eBioscience, 16-0288-81) for 24 h, we started cells collection. Besides, sodium oxamate is the key enzyme of glycolysis which could enhance the hypoxic microenvironment by inhibiting glycolysis. BxPC-3 cells washed by PBS were pretreated with 50 mm sodium oxamate (Abcam) for 24 h and then washed before co-cultivation. Lactic acid (Tokyo Chemical Industry Co., Ltd.) was directly added into the co-culture. We used a three-gas incubator (MiniGalaxy A; RS Biotech) to create Hypoxic conditions (1% O_2_) by injection of N_2_ (1% O_2_/94% N_2_/5% CO_2_ atmosphere) at 37 °C.

### Peripheral blood mononuclear cell (PBMC) preparation

Blood samples came from donors after the provision of written informed consent. A total of 2 ml heparin-anticoagulated venous blood was diluted and then gently added onto 4 ml Lympholyte®-H Cell Separation Media (Cedarlane®), then ccentrifugated for 20 min (2000 rpm, room temperature). After washed with PBS, we centrifuged three times at 1500 rpm at room temperature again. The pellets were resuspended in 1 ml RMPI 1640 to obtain a PBMC suspension.

### Isolation of CD4^+^ T cells

According to the Isolation Kit (MiltenyiBiotec, Inc.), we isolated the CD4^+^ T cells. Resuspended 1 × 10^7^ PBMCs in 40 µl PBS (supplemented with 0.5% FBS), labeled with 10 µl CD4^+^ T-Cell Biotin-Antibody Cocktail for five min at 4 °C, 20 µl CD4^+^ T-Cell MicroBead Cocktail for 10 min at 4 °C. After centrifuged, we resuspended in 500 µl PBS supplemented with 0.5% FBS. Then separated CD4^+^ T cells by LS Separation columns and incubated in RMPI 1640 supplemented with 10% FBS, 100 U/ml penicillin, and 100 U/ml streptomycin. Flow cytometric analysis was used to detect the purity of the CD4^+^ T cells.

### Lactic acid detection

Lactic acid was detected by a lactate assay kit (Nanjing Jiancheng Bioengineering Institute, A019-2). After mixed samples with a working solution containing LDH, we then incubated in a 37 °C water bath for 10 min. The optical density (O.D.) values were obtained using the GloMax®-Multi + Detection System (Promega Corporation) at a wavelength of 530 nm. Distilled water was used as the control.

### Determination of glucose uptake ability

After washed with glucose-free Krebs–Ringer bicarbonate buffer supplemented with 2% BSA, T cells was then been incubated with glucose-free RMPI 1640 containing 100 μg/ml 2-[*N*-(7-nitrobenz-2-oxa-1,3-diaxol-4-yl) amino]-2-deoxyglucose (37 °C, 60 min). Then, digested via trypsinization and followed by immediate assessment of fluorescence intensity using the GloMax®-Multi + Detection System (Promega Corporation) at a wavelength of 485 nm.

### ATP assay

CD4^+^ T cells were seeded and incubated for 96 h prior to harvesting. ATP was measured by ENLITEN ATP Assay System (Promega Corporation).

### Detection of cellular viability and proliferation

After incubation for 0, 24, 48, 72, 96, or 120 h, the viability of CD4^+^ T cells was detected by Cell Counting Kit-8 (CCK-8) assay. O.D. values were determined using the GloMax®-Multi + Detection System (Promega Corporation) at a wavelength of 450 nm.

### Detection of apoptosis

After digested via trypsinization, the cells were then counted. Then, 5–10 × 10^4^ resuspended cells were centrifuged (1000×*g*, 37 °C, five min). The supernatant was aspirated, and 195 μl binding buffer was added to the pellet, stained with 5 μl Annexin V-FITC at 4˚C and then added 5 µl propidium iodide, incubated for another five min under the same conditions. Apoptosis was then evaluated by flow cytometry (BD, USA).

### Reverse transcription-quantitative (RT-q) PCR

After isolated from the CD4^+^ T cells by TRIzol® reagent (Invitrogen, USA), the total RNA was stored in DEPC-treated water at − 80 °C. For usage, DNA was digested by DNase I treatment (37 °C, 30 min). After denatured at 65 °C. for five min, the purified RNA then immediately cooled on ice. After reverse transcribed by RevertAid First Strand cDNA Synthesis Kit, the qPCR was performed by SYBR Green PCR kit on a Step One Plus™ Real-Time PCR System.

The following primers:

CD3: forward,3’-ATGAGCTGTGCACAAAGTGG-5’ and reverse,3’-ACATTGACG GGTTTTTCCTG-5’;

CD28: forward,3’-CAGCAGTACTTGGGTGCTGA-5’ and reverse,3’-TATTTG CCACTGCCATTTCA-5’;

CD25: forward, 3’-AGCGGAGACAGAGGAAGAG-5’ and reverse,3’-GGCAAG CACAACGGATG-5’;

CD69: forward,3’-GTGCTGTAATGAATGTGGTC-5’ and reverse,3’-GTAGCATT TCCTCTGGTAGCC-5’;

CD71: forward,3’-ATGATGGATCAAGCTAGATCAGCAT-5’ and reverse,3’-TTG GTTTTGTGACATTGGCCT-5’;

HLA-DR: forward,3’-GCCTCTTCTCAAGCACTGGGA-5’ and reverse,3’-CCA CCAGACCCACAGTCAGG-5’;

ZAP70: forward,3’-CATGAGTGACTGCTGGATCTACAA-5’ and reverse,3’-GCT GGCCAGGCTGTAGTAACA-5’;

GAPDH: forward,3’-CACATGGCCTCCAAGGAGTAA-5’ and reverse,3’-TGAGG GTCTCTCTCTTCCTCTTGT-5’.

### Detection of cellular markers

CD4^+^ T cells were incubated with human CD3 (cat. no. APC-65060; ProteinTech Group, Inc.), CD28 (cat. no. #62-0289-42; eBioscience; Thermo Fisher Scientific, Inc.), CD25, and CD69 (cat. no. FAB1020A and FAB23591P, respectively; R&D Systems, Inc.), and CD71 and HLA-DR (cat. no. ab9179 and ab20181, respectively; Abcam) antibodies (4˚C). FlowJo software (FlowJo version 7 LLC) was used for data analysis.

### Western blot analysis

After concentrated the cell lysates and resolved by SDS-PAGE, the proteins were then transferred to polyvinylidene fluoride membranes and immunoblotted. The density of each band was measured using ImageQuant LAS 500 software (Cytiva). The antibodies included primary antibodies against DKK3 and GAPDH (cat. no. ab126080 and ab181602, respectively; Abcam); active de-phospho-β-catenin, E-cadherin, N-Cadherin, and LDHA (cat. no. #19,807, #14,472, #14,215 and #3582, respectively; Cell Signaling Technology, Inc.); GLUT1 and PDK1 (cat. no. sc-377228 and sc-17765, respectively; Santa Cruz Biotechnology, Inc.); HIF-1α and HK2 (cat. no. AP1029b and AM8606b, respectively; Abgent, Inc.); and secondary horseradish peroxidase-conjugated antibodies (Beyotime Institute of Biotechnology). Western blot analysis of OXPHOS in mitochondria isolated from pancreatic cancer cells was performed using an OXPHOS Monoclonal antibody cocktail (cat. no.# 45–8199, Invitrogen; Thermo Fisher Scientific, Inc.).

### ELISA

The concentrations of multiple cytokines were determined using the ELISA method. The ELISA Kit were purchased from R&D Systems, Inc (RDsystem (Duoset Elisa DY317-05) and RD system (DIF50)). The samples were assessed within 30 min using a DNM-9602 microplate reader (Prolong, Beijing, China), and the data were presented as concentrations (pg/ml).

### Statistical analysis

SPSS 22.0 was used for data analysis. Continuous data that obey the normal distribution were expressed as mean values ± standard deviation; quantitative data that do not follow a normal distribution are represented by the median (interquartile range) [M(P25, P75)]. Comparisons among multiple groups was tested by one-way analysis of variance. A *P* < 0.05 was considered significant difference.

## Results

### DKK3 inhibits aerobic glycolysis in pancreatic cancer BxPC-3 cells

**Fig. 1:**
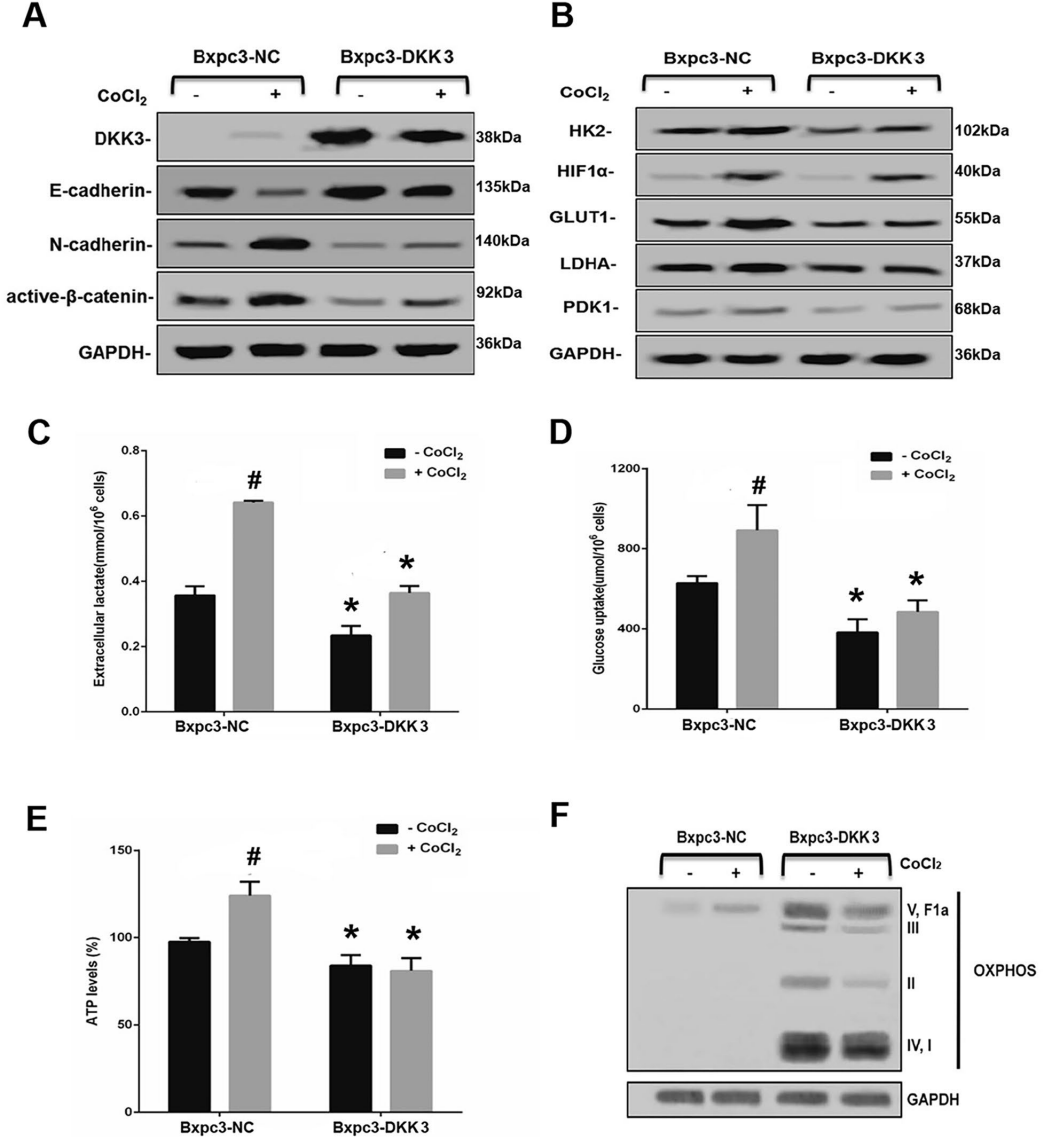
DKK3 inhibits aerobic glycolysis. **A** Western blot analysis of epithelial–mesenchymal transition markers underhypoxic conditions. **B** Western blot analysis of aerobic glycolysis markers under hypoxic and normoxic conditions. Levels of **C** extracellular lactic acid, **D** glucose uptakeand, **E** ATP production. **F** OXPHOS protein expression in mitochondria isolated from pancreatic cancer cells. D ^#^*P* < 0.01: compared with the BxPC-3 NC (–CoCl_2_) group; ^*^P < 0.01: compared with the BxPC-3 NC group. DKK3, dickkopf-related protein 3; OXPHOS, oxidative phosphorylation

Compared with normal control groups, the expression of DKK3 protein increased in the DKK3-overexpression group (Fig. 1A). Western blot analysis showed upregulated expression of aerobic glycolysis markers (HK2, HIF-1α, GLUT-1, LDHA, and PDK-1) in the NC group under hypoxic conditions. In DKK3-overexpressing cells, the phenomenon was reversed under normal and hypoxic conditions, indicating that DKK3 negatively regulates aerobic glycolysis (Fig. 1B). The levels of extracellular lactate (Fig. 1C), glucose uptake (Fig. 1D), and ATP production (Fig. 1E) in the NC group were remarkably increased under hypoxic conditions. However, these levels were significantly lower in DKK3-overexpressing cells than NC group in both hypoxic and normoxic conditions, indicating that DKK3 inhibits aerobic glycolysis regardless of the presence of hypoxia. Furthermore, the expression of OXPHOS significantly promoted whether under hypoxic conditions or not (Fig. 1F).

### Effects of DKK3-overexpression on glucose, proliferation, and apoptosis in CD4^+^ T cells

**Fig. 2:**
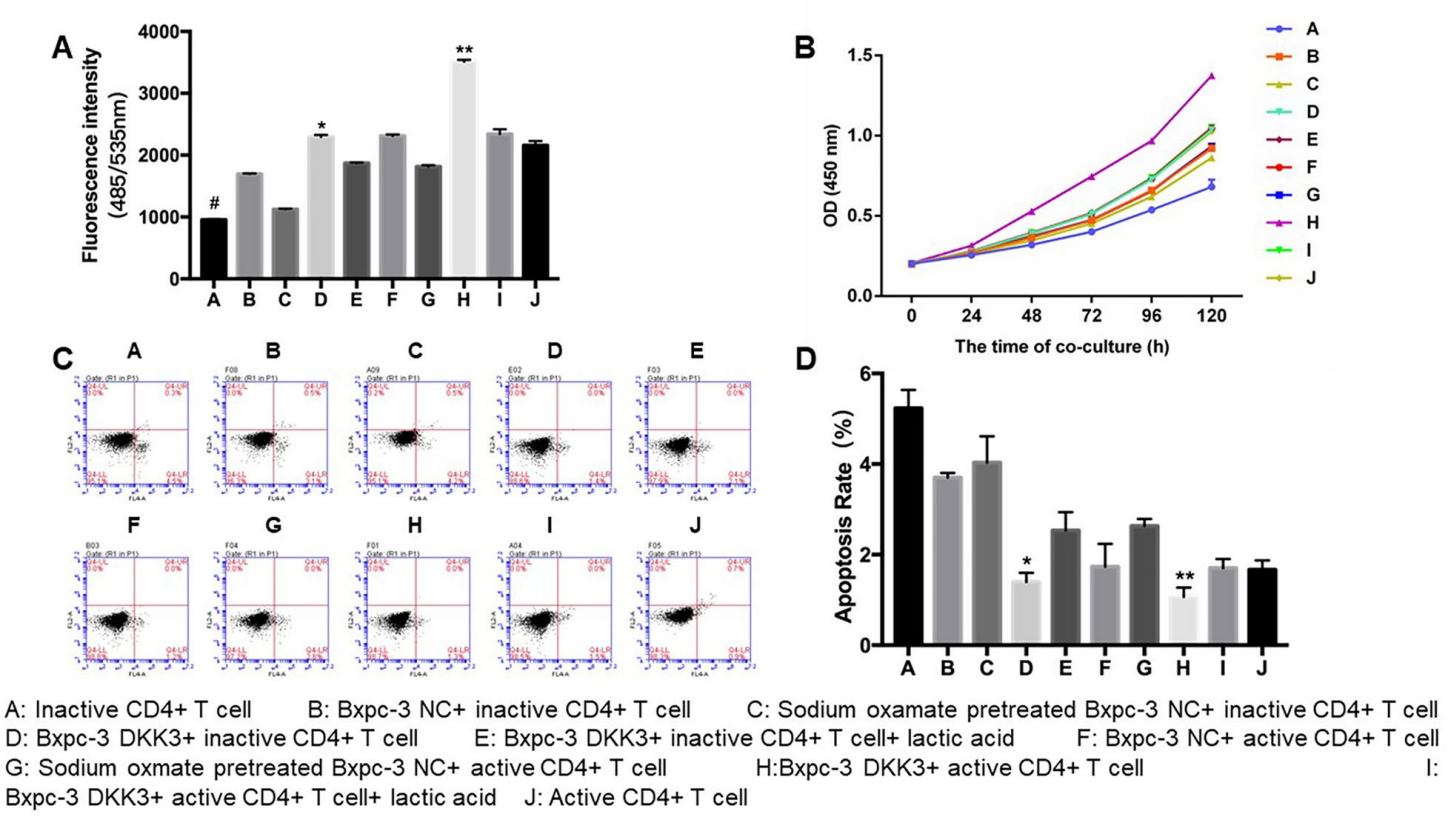
Effects of DKK3-overexpression in pancreatic cancer on CD4^+^ T-cell glucose levels, proliferation, and apoptosis. **A** Levels ofglucose uptake in CD4^+^ T cells. **B** Proliferative activity of CD4^+^ T cells. **C**, **D** Proportions of apoptotic cells by flow cytometric analysis. ^#^*P* < 0.01: compared with groups B, C and D. ^*^P < 0.01: compared with groups B and E. ^**^P < 0.01 compared with groups F and I. DKK3, dickkopf-related protein 3

Glucose levels in inactive co-cultured CD4^+^ T cells were markedly increased than those of inactive CD4^+^ T cells only. Furthermore, the glucose levels in inactive CD4^+^ T cells co-cultured with DKK3-overexpressing pancreatic cancer BxPC-3 cells were higher than normal control group. The glucose level in activated CD4^+^ T cells was also increased under co-culture conditions, especially in the DKK3-expression group. However, the glucose uptake level in the activated CD4^+^ T cells that were co-cultured with sodium oxamate-pretreated BxPC-3 cells was significantly less than control group (Fig. 2A). The results of ACCK-8 assay showed that the proliferative level of each group was higher than normal control group.

Moreover, the proliferative activity of CD4^+^ T cells in Group H (activated CD4^+^ T cells co-cultured with DKK3-expressing BxPC-3 cells) was higher than activated CD4^+^ T cell only group (Fig. 2B). As shown in Fig. 2C, D, the level of apoptosis in the DKK3-expressing co-cultured group was other groups. Therefore, co-culturing with DKK3-expressing BxPC-3 cells promotes glucose uptake, enhances proliferation, and inhibits apoptosis in CD4^+^ T cells.

### Regulatory effects of DKK3-overexpression on the activation of CD4^+^ T cells

**Fig. 3:**
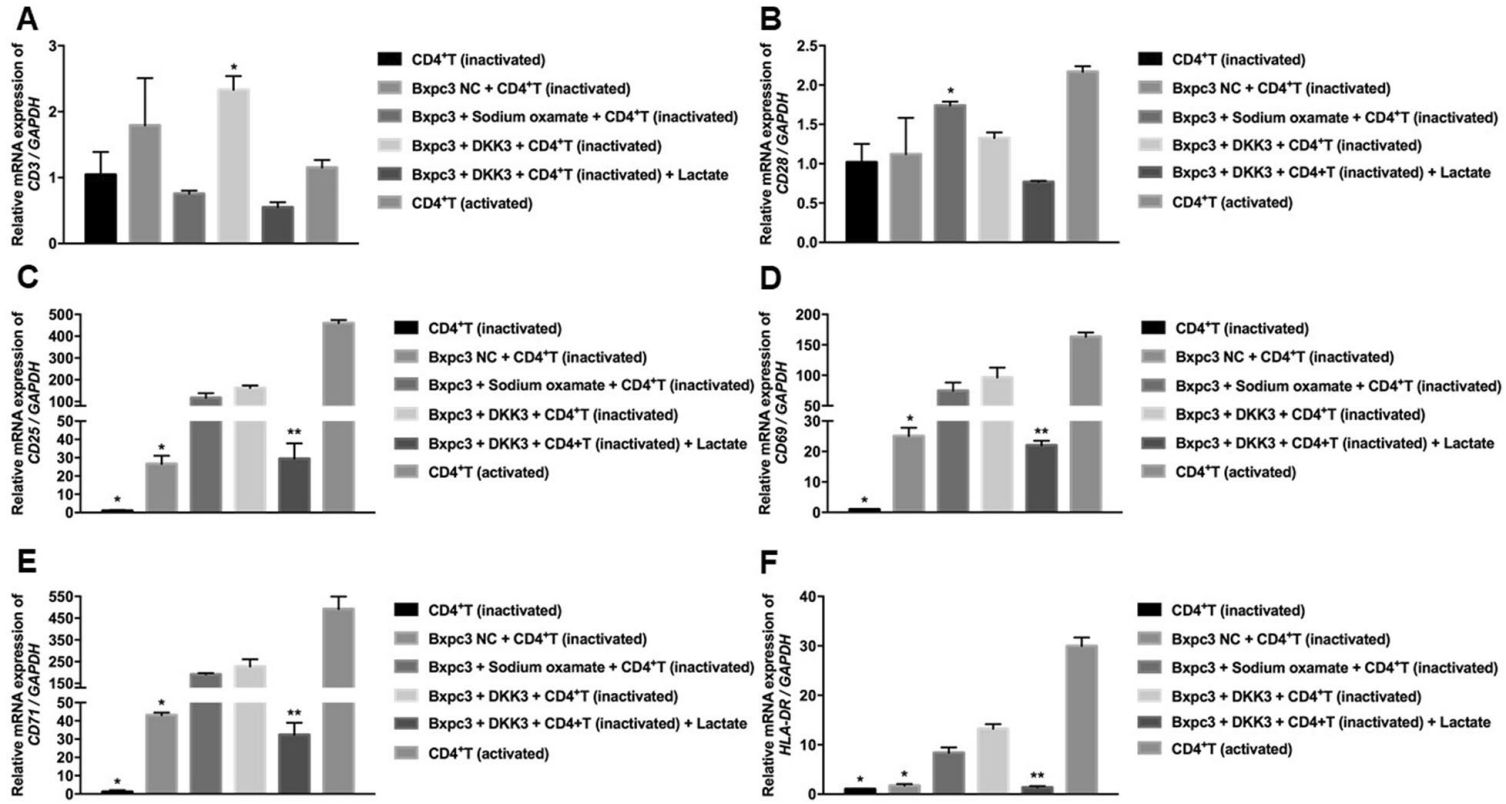
Relative mRNA expression levels of **A** CD3, **B** CD28, **C** CD25, **D** CD69, **E** CD71 and **F** HLA-DR in inactivated CD4^+^ T cells of each group after 72 h of co-culture. DKK3, dickkopf-related protein 3; NC, negative control

Relative to the other groups, co-culturing BxPC-3-DKK3 cells with inactivated CD4^+^ T cells upregulated the relative mRNA expression of CD3 in the latter (Fig. 3A). However, only co-culturing sodium oxamate-pretreated BxPC-3 cells with inactivated CD4^+^ T cells promoted an increase in the relative mRNA expression of CD28 (Fig. 3B). The relative mRNA expression levels of CD25 and CD69 were significantly increased in inactivated CD4 + T cells co-cultured with BxPC-3-DKK3 cells. As shown in Fig. 3C, D, CD25 and CD69 mRNA increased in CD4^+^ T cells co-cultured with sodium oxamate when compared with the corresponding untreated group.

Furthermore, the levels of CD25 and CD69 mRNA in lactic acid treatment group were markedly lower than DKK3-overexpression group (Fig. 3C, D). The percentages of CD4^+^ T-cell CD71 and HLA-DR mRNA in co-cultures treated with sodium oxamate group were significantly higher control group. DKK3-overexpression also promoted the expression of CD71 and HLA-DR mRNA, while lactic acid reduced the effects of DKK3, which was similar to the results of CD25 and CD69 detection (Fig. 3E, F).

**Fig. 4:**
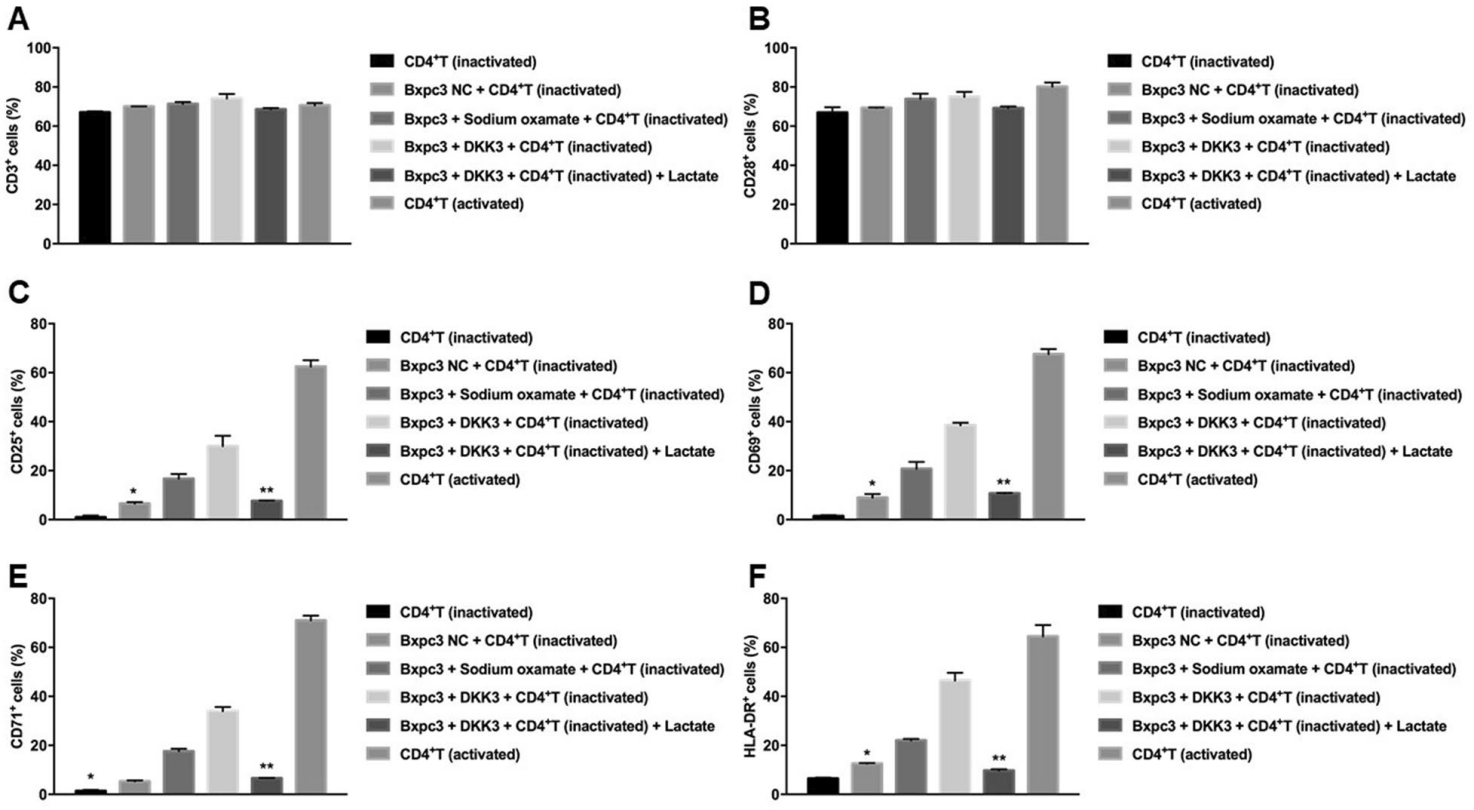
Flow cytometric detection of inactivated CD4^+^ T-cell surface markers. Proportions of **A** CD3^+^, **B** CD28^+^, **C** CD25^+^, **D** CD69^+^, **E** CD71^+^ and **F** HLA-DR^+^ cells. ^*^P < 0.01: compared with the sodium sulphate-pretreated BxPC-3 NC group and BxPC-3-DKK3 group; ^**^ *P* < 0.01: compared with the BxPC-3-DKK3 group. Data are presented as the mean ± SE of three independent experiments. DKK3, dickkopf-related protein 3; NC, negative control

Unlike the results of RT-qPCR, flow cytometry showed that the proportion of CD3^+^ and CD28^+^ cells were similar among groups (Fig. 4A, B). However, the percentage of CD25^+^, CD69^+^, CD71^+^, and HLA-DR^+^ cells in inactivated CD4^+^ T cells co-cultured with BxPC-3-DKK3 cells group was significantly higher than BxPC-3NC group. Moreover, sodium oxamate-pretreated BxPC-3 NC cells showed a significantly higher proportion of CD25^+^, CD69^+^, CD71^+^, and HLA-DR^+^ cells than BxPC-3 NC cells co-cultured with inactivated CD4^+^ T cells. However, the percentages of cells expressing these markers were significantly reduced following the addition of lactic acid to the BxPC-3-DKK3/inactivated CD4^+^ T-cell co-culture group (Fig. 4C–F).

### Overexpression of DKK3 induces INF-γ, IL-2, IL-17, ZAP70, and suppresses IL-10 and IL-4 expression

**Fig. 5:**
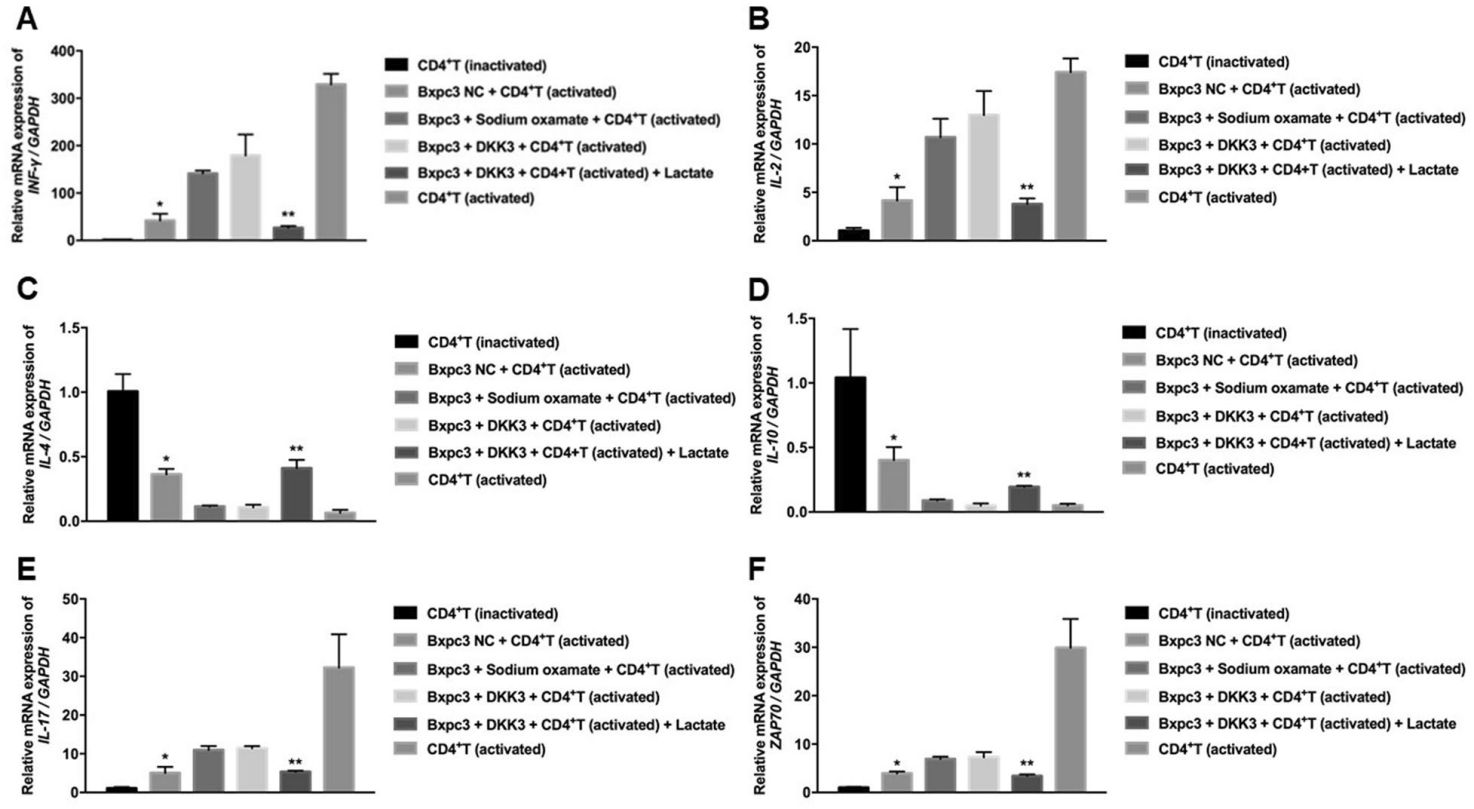
Relative mRNA expression levels of **A** IFN-γ, **B** IL-2, **C** IL-4, **D** IL-10, **E** IL-17 and **F** ZAP70 in activated CD4^+^ T cells of each group after 72 h of co-culture. ^*^P < 0.01: compared with the sodium sulphate-pretreated BxPC-3 NC group and BxPC-3-DKK3 group; ^**^P < 0.01: compared with the BxPC-3-DKK3 group. DKK3, dickkopf-related protein 3; NC, negative control

The results showed that co-culture with BxPC-3-DKK3 cells enhanced the relative expression of IFN-γ, IL-2, IL-17, and ZAP70 mRNA, reduced the level of IL-4 and IL-10 in activated CD4^+^ T cells. Due to its ability to inhibit aerobic glycolysis and reduce the production of lactic acid, BxPC-3 cells were pretreated with sodium oxamate, which also upregulated the expression of IFN-γ, IL-2, IL-17, and ZAP70, as well as downregulating the level of IL-4 and IL-10 in activated CD4^+^ T cells. Moreover, lactic acid treatment reversed the effects of DKK3 on IFN-γ, IL-2, IL-17, ZAP70, IL-4, and IL-10 mRNA in activated CD4^+^ T cells (Fig. 5).

**Fig. 6:**
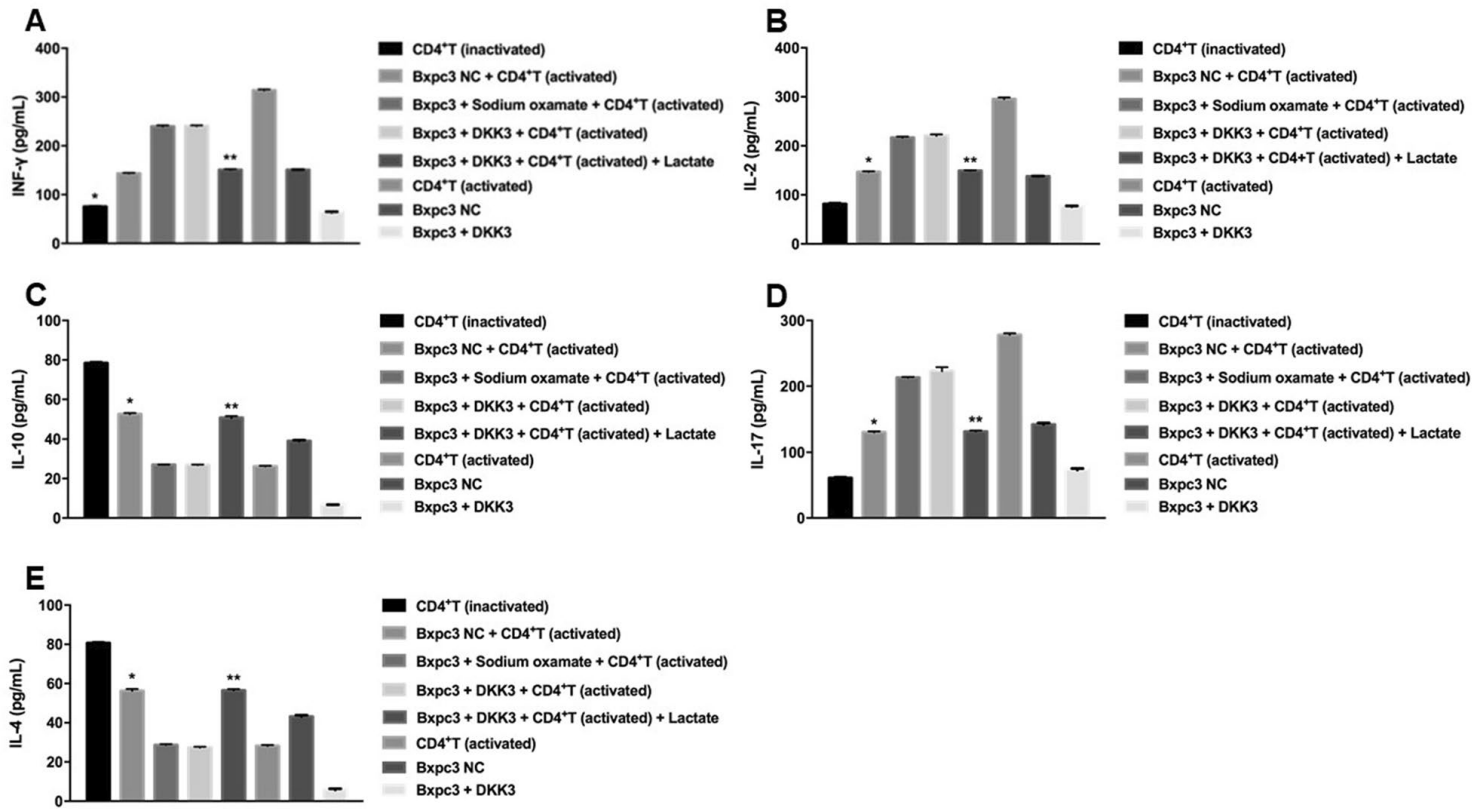
Concentrations of **A** IFN-γ, **B** IL-2, **C** IL-10, **D** IL-17 and **E** IL-4 in the supernatants of activated CD4^+^ T cells co-culturedfor 96 h, detected by ELISA. ^*^P < 0.01: compared with the sodium sulphate-pretreated BxPC-3 NC group and BxPC-3-DKK3 group; ^**^P < 0.01: compared with the BxPC-3-DKK3 group.DKK3, dickkopf-related protein 3; NC, negative control

IFN-γ, IL-2, and IL-17 levels were significantly higher in the sodium oxamate-pretreatment and DKK3-overexpression groups than normal control group. However, the concentrations of IL-4 and IL-10 were decreased by sodium oxamate or DKK3-overexpression. Furthermore, lactic acid treatment reversed the effects of DKK3 on IFN-γ, IL-2, IL-17, IL-4, and IL-10 in activated CD4^+^ T-cell supernatants (Fig. 6).

## Discussion

**Fig. 7:**
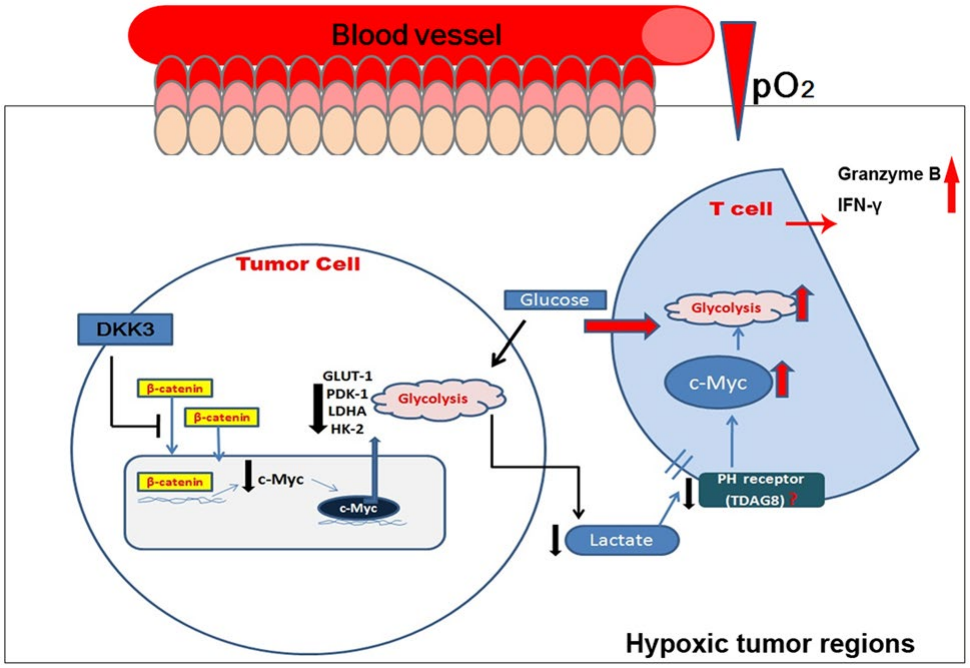
DKK3 inhibits aerobic glycolysis in pancreatic cancer BxPC-3 cells, which promotes glucose uptake, enhances proliferation, and inhibits apoptosis in CD4^+^ T cells

Malignant transformation of cells requires enhanced glucose and glutamine consumption rates, which are transformed into ATP for growth, differentiation, and proliferation [3]. Wnt/β-catenin signaling is important in tumor initiation and progression [9, 10]. DKK3 may be involved in Wnt pathway regulation [11]. Our preliminary trial found that DKK3 is significantly downregulated in BxPC-3 cells and suppresses the epithelial–mesenchymal transition of these cells under hypoxic conditions [8]. But the specific mechanisms of DKK3 in treating pancreatic cancer remains unknown [12, 13]. The associative diagram showing the relationship between DKK3 in Wnt signaling, T-cell activation markers, cytokines, etc., is presented in Fig. 7.

The Wnt/β-catenin signaling pathway played an important role in tumor cell functions [14, 15]. Our study demonstrated a mechanism by which DKK3 inhibits aerobic glycolysis in BxPC-3 cells by downregulating the glycolysis markers HK2, HIF-1α, GLUT-1, LDHA, and PDK-1, as well as the levels of extracellular lactate and glucose uptake. ATP production was also decreased in the DKK3-expression group, suggesting that aerobic glycolysis is inhibited by DKK3 whether under hypoxic or normoxic conditions. Inhibiting aerobic glycolysis in BxPC-3 cells can delay the growth, proliferation, and differentiation of tumor cells. Metabolic changes in BxPC-3 cells are critical for CD4^+^ T-cell dysfunction by promoting glucose uptake therein [16, 17].

Furthermore, lactic acid is one of the most important products of aerobic glycolysis in tumor cells [18]. Previous study [19] found that lactic acid could promoted tumor development by suppressing T-cell activation and proliferation. In the present study, inhibiting tumor cell glycolysis resulted in a decrease in lactic acid production, which indicated that DKK3 maybe enhanced the activation and proliferation of T cells by inhibiting tumor cell glycolysis.

Specific immune cells expressing the Wnt receptor may be therapeutic targets for proteins, such as DKK3 [20]. Additional studies have confirmed the immunomodulatory role of DKK3, i [6]. However, in the present study, DKK3 increased the glucose level, promoted the proliferation of CD4^+^ T cells and decreased apoptosis levels. The pathways associated with oxidative metabolism promote the effector functions of immune cells [21–24]. Similar results were presented in another study, where T cells were shown to use glucose as the primary nutrient for ATP production, which is essential for activation, proliferation, and cytokine generation [2]. Above all, DKK3 can increase glucose metabolism of CD4^+^ T cell and reduce the level of apoptosis. In support of our previous studies and other researchers’ findings, DKK3 was confirmed to inhibit BxPC-3 cell proliferation and induce tumor cell apoptosis [8, 25, 26].

Besides, our study also revealed that co-culturing with DKK3-overexpressing pancreatic cancer cells promotes the expression of CD69, CD3, CD25, CD71, and HLA-DR mRNA in CD4^+^ T cells. Moreover, pretreatment with sodium oxalate also promoted the expression of cytokines. In the past few decades, similar and contradictory conclusions have been obtained [27–30] that precisely reflect the subtle characteristics of Wnt/β-catenin signaling. The effects of suppressing Wnt/β-catenin signaling in CD4^+^ T cells have been observed in several studies [28, 31, 32]. Furthermore, our study found that co-culture with DKK3-overexpressing pancreatic cancer cells upregulated IFN-γ, IL-2, and IL-17, inhibited tIL-4 and IL-10 in activated CD4^+^ T cells. Therefore, DKK3 maybe enhanced CD4^+^ T-cell-mediated anti-BxPC-3 cell immunity. This may be due to the fact that the effects of DKK3 differ between distinct subpopulations of CD4^+^ T cells.

Previous study reported that the suppression of Wnt/β-catenin signaling promoted the expression of IL-2 and IL-17 in activated CD4^+^ T cells [3, 33, 34]. Therefore, DKK3 may enhance Th1 and Th17 cell-mediated BxPC-3 cell death. In the present study, the production of signature Th2-associated cytokines reduced in the DKK3-overexpression group. Thus, these results establish DKK3 promoted Th1 and Th17 cell differentiation while retarding Th2 differentiation in activated CD4^+^ T cells.

The current study found that lactate inhibits the excitation of CD4^+^ T cells and inhibits Th1, Th17, and IFN-γ differentiation, promoted Th2 cell differentiation in activated CD4^+^ T cells. A previous study also reported that lactic acid inhibited T-cell receptor-triggered cytokine production impaired lytic granule exocytosis in cytotoxic T cells [35]. Thus, lactic acid plays a crucial immunoregulatory role in pancreatic cancer. Furthermore, sodium oxamate, an inhibitor of LDH, can suppress pancreatic cancer cell proliferation; thus, LDH is the target of several currently used cancer therapies [36].

However, the main limitation of this study is that there is no in vivo experiment to verify the results of the study. Therefore, animal experiments can be added in future studies for further verification.

## Conclusions

DKK3 alters the metabolic microenvironment of pancreatic cancer cells and further facilitates the function of CD4^**+**^ T cells which suggesting that DKK3 may have a therapeutic potential in pancreatic cancer.

## Data Availability

All data generated or analyzed during this study are included in this published article.
